# Collective health research assessment: developing a tool to measure the impact of multistakeholder research initiatives

**DOI:** 10.1186/s12961-022-00856-9

**Published:** 2022-05-02

**Authors:** Anna-Aurora Kork, Carla Antonini, Nicolás García-Torea, Mercedes Luque-Vílchez, Ericka Costa, Juliette Senn, Carlos Larrinaga, Deborah Bertorello, Giampaolo Brichetto, Paola Zaratin, Michele Andreaus

**Affiliations:** 1 Faculty of Social Sciences, Tampere University, Tampere, Finland; 2 Department of Accounting, Universidad Autónoma de Madrid, Madrid, Spain; 3 Department of Economy and Business Administration, Universidad de Burgos, Burgos, Spain; 4 Department of Agriculture Economy, Finance and Accounting, Universidad de Córdoba, Córdoba, Spain; 5 European Financial Reporting Advisory Group (EFRAG), Brussels, Belgium; 6 Department of Economic and Management, University of Trento, Trento, Italy; 7 Montpellier Business School, Montpellier, France; 8 FISM-Italian Multiple Sclerosis Society Foundation, Genoa, Italy

**Keywords:** Research impact, Measurement, Scorecard, Multistakeholder, Patient-reported dimension, Responsible research and innovation, Payback

## Abstract

**Background:**

The need to more collaboratively measure the impact of health research and to do so from multidimensional perspectives has been acknowledged. A scorecard was developed as part of the Collective Research Impact Framework (CRIF), to engage stakeholders in the assessment of the impacts of health research and innovations. The purpose of this study was to describe the developmental process of the MULTI-ACT Master Scorecard (MSC) and how it can be used as a workable tool for collectively assessing future responsible research and innovation measures.

**Methods:**

An extensive review of the health research impact literature and of multistakeholder initiatives resulted in a database of 1556 impact indicators. The MSC was then cocreated by engaging key stakeholders and conducting semi-structured interviews of experts in the field.

**Results:**

The MSC consists of five accountability dimensions: excellence, efficacy, economic, social and patient-reported outcomes. The tool contains 125 potential indicators, classified into 53 impact measurement aspects that are considered the most relevant topics for multistakeholder research and innovation initiatives when assessing their impact on the basis of their mission and their stakeholders’ interests. The scorecard allows the strategic management of multistakeholder research initiatives to demonstrate their impact on people and society. The value of the tool is that it is comprehensive, customizable and easy to use.

**Conclusions:**

The MSC is an example of how the views of society can be taken into account when research impacts are assessed in a more sustainable and balanced way. The engagement of patients and other stakeholders is an integral part of the CRIF, facilitating collaborative decision-making in the design of policies and research agendas. In policy making, the collective approach allows the evaluation perspective to be extended to the needs of society and towards responsible research and innovation. Multidimensionality makes research and innovations more responsive to systemic challenges, and developing more equitable and sustainable health services.

**Supplementary Information:**

The online version contains supplementary material available at 10.1186/s12961-022-00856-9.

## Background

Concerns have recently been raised about the social impact and ethical considerations of research. In health research, studies have identified a high level of dissatisfaction with how research is assessed and how its effects are mostly defined in academic or economic terms [1–3]. Specifically, the traditional assessment of health research on the basis of academic outputs is, arguably, linked with (i) a lack of translation into healthcare policy and practice [4, 5] and (ii) ineffective use of scarce resources in both research and health systems [6]. Therefore, assessing the impact of research beyond traditional measures is crucial [7, 8]. The search for new measures gives prominence to ethical issues [9] and the needs of patients and society [10].

Previous research has revealed a need for a paradigm shift in the measurement of the impacts of health research towards more collaborative and multidimensional forms of assessment. This could be, for example, engaging a wide array of stakeholders [11] and considering multicultural environments [12] when collectively defining impact assessment systems [10, 13]. To this end, the European Commission [14] has proposed the responsible research and innovation (RRI) approach for making research more inclusive, responsive and sustainable. The key principles of RRI are the promotion of public engagement, gender equality, ethics, science education and open access in research governance [15]. Despite the relevance attributed to RRI for enhancing the democratic governance of research and innovation, the need to clarify the arguments behind RRI policies putting practices in place has been acknowledged [16–18]. Overall, RRI means a wide variety of stakeholders working together to align both research processes and outcomes with the values, needs and expectations of society [17–20]. However, turning the ideals of bottom-up engagement or citizen involvement into institutional action [21], has proven challenging. The most problematic element of implementing RRI might be the “interactive model of the research utilization process” [22], that is, a multiplicity of related interests and actors interacting and communicating the benefits or outcomes of research. A considerable number of studies have focused on developing frameworks for implementing RRI (see e.g. [17, 23, 24]), but tools are lacking for assessing whether and how research and innovations actually follow RRI principles in line with the needs of society [25], or placing the patient perspective at the core of the impact assessment [26].

Hence, to address these needs, this paper presents a framework with a tool that facilitates the operationalization of the RRI approach. The Collective Research Impact Framework (CRIF) was developed in the MULTI-ACT project to go beyond the traditional forms of research assessment and to increase the impact of health research on patients and society. The premise of the development lies in the observation that sustainable research needs a transformational mission and collective impact assessment [13]. CRIF is a holistic concept, encompassing a governance model, patient engagement guidelines and a multidimensional impact assessment. The key element of its impact assessment is the Master Scorecard (MSC), which provides a collection of indicators, classified under five impact dimensions. The MSC was designed as part of the CRIF to engage multiple stakeholders in a collective impact assessment. Instead of single measures determined by individual organizations, tackling the needs of patients and society requires collective action and a shared mission defined by all the relevant stakeholders of health research. This innovative co-accountability strategy aims to promote participatory and anticipatory governance in health research (see [26]) and to respond to the different demands of stakeholders by enabling the alignment of a plurality of interests in a shared mission.

**Fig. 1:**
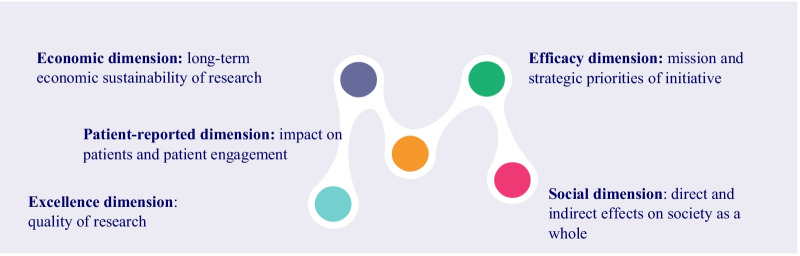
Interrelated impact measurement dimensions in CRIF

To be able to solve “grand challenges” such as environmental or social sustainability, demographic change or complex health problems, RRI requires a direction that enables mission-oriented and multistakeholder governance models as these ensure cooperation and the pursuit of a long-term return on investment that is not only economic (e.g. [26–28]). In this respect, one of the innovations of CRIF is that it considers mission orientation to be an explicit driver of accountability and introduces the first accountability dimension (efficacy), defined as the capacity of a research initiative to fulfil the shared mission. The second accountability dimension (excellence) posits that the development of high-quality health research must be aligned with the mission of health research. Further CRIF accountability dimensions assess the co-participation of all the stakeholders who directly or indirectly participate in the field (social), while enabling financial sustainability (economic). The fifth dimension related to the impacts on patients and people affected by a disease (patient-reported outcomes [PRO]) is transversal, to be applied across the other four dimensions. It highlights the active engagement of stakeholders throughout the research process. Figure 1 shows the five accountability dimensions of the CRIF that reflect the interrelated perspectives of impact measurement: excellence, efficacy, economic, social and PROs.

So far, most of the conventional research impact assessment frameworks for measuring the impact on people or health have not been sufficiently effective and applying them in multistakeholder initiatives has been challenging, as they may lack shared impact measures, a broader picture of impacts or supporting infrastructure to allow the involvement of multiple stakeholders for the true alignment of efforts and the accountability of results (see e.g. [29–31]). Although progress is being made, the operationalization of impact is still underdeveloped if research-related contribution and the alignment of efforts are not assessed [32]. These shortcomings have narrowed down the measurement of health research impacts and discouraged the true commitment of the various stakeholders to being co-accountable for the results. To overcome these issues, the MSC was developed as a workable tool for collaborative decision-making when assessing the return on investment of research that best reflects the shared mission and the relevant claims of each stakeholder, including benefits for patients and society.

The MSC can be applied at the beginning of or during a research project to engage multiple stakeholders in collectively defining the impacts and selecting the indicators of a given mission. Therefore, to achieve maximum impact, effective collaboration and dialogue is needed among all stakeholders involved in health research and innovation, such as patient organizations, academics, funders, policy-makers, healthcare organizations, and pharmaceutical and biotechnological companies. With the MSC, the purpose of this collective evaluation is to consider the diversity of the stakeholders’ interests as well as to reflect the variety of impact dimensions and measurement aspects of health research impacts. The multidimensionality of impact evaluation is based on a broad understanding of the return on investment. This means that the needs and benefits of different stakeholders such as patients and their caregivers are considered when assessing the returns on research rather than focusing purely on academic or short-term financial outcomes. By aligning the divergent priorities of different stakeholders, the collective evaluation process enables co-accountability among all the relevant stakeholders, to progress towards the shared mission for the benefit of society as a whole [33, 34].

The contribution of this paper is twofold. First, the study responded to the call to go beyond conventional metrics when assessing the impact of health research [3, 10] and to implement RRI [16, 17], particularly by providing multidimensionality and linking collaborative actions to impact assessment. The MSC has the potential to accomplish this task because it contains over 100 indicators from different dimensions for multiple accountability purposes that are important both in RRI (excellence, economic, social) and for monitoring PROs and the shared mission (efficacy) to enhance mission-oriented multistakeholder health research [34, 35]. Second, by describing how MSC can be used, this article complements the recent study by Zaratin et al. [26] that introduces the CRIF and its holistic management model for fostering RRI in brain research. By going into one element of this framework in more detail, this study focused on the MSC development process, the multistakeholder nature of which is consistent with RRI. It combined expertise in different fields with literature reviews, individual interviews, and focus groups of experts, patient organizations and practitioners.

The remainder of the paper is organized as follows. The next section lays out the methods and the key developmental phases of the MSC. The “Results” section presents the results of the analysis and the advanced functionalities and multidimensional impact indicators of MSC. The paper concludes with a discussion on the potential value and the usability of this kind of collective assessment tool for implementing and assessing the RRI measures of future multistakeholder initiatives.

## Methods

The development of the MSC was guided by the strategic intent of responsible research and key building principles of the CRIF: (i) participatory governance, (ii) effective and inclusive stakeholder engagement, (iii) shared mission and agenda, and (iv) collective measurement for the bottom-up evaluation of health research impacts that advance collaboration between science and society and integrate scientific excellence into patients’ needs and social responsibility [26]. To develop a formal tool for assessing the impact of health research, we focused on impacts rather than performance per se, as it is impact that makes research transformative. This project defined impact as the long-term socioeconomic changes brought about by the intervention (i.e. over 5 years), and the impact indicators as means for measuring the achievement, performance and changes connected to the intervention.

**Fig. 2:**
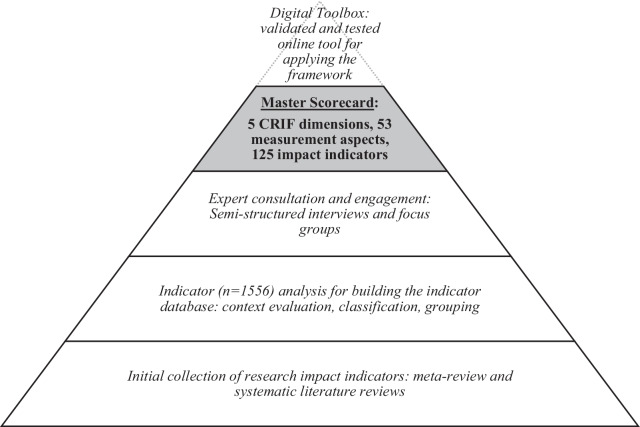
Key phases in development process of MULTI-ACT MSC

The MSC was accomplished as part of the multistakeholder engagement process of the MULTI-ACT project. First, we built an initial database of the health research impact indicators through a meta-review and systematic literature review to explore the existing health research impact frameworks and indicators already being used in the health sector and in multistakeholder organizations. Second, we conducted in-depth analysis and classification of the indicators to understand their underlying rationale in terms of the setting in which they were proposed and to evaluate their alignment with the CRIF. Third, as the engagement of various stakeholders lies at the core of RRI and the framework, we identified key stakeholders to adopt a multistakeholder cocreation approach for gathering their perceptions to refine the selection of the most suitable indicators. The data collection and analysis methods are described below (see Fig. 2).

### Initial collection of impact indicators: meta-review and systematic literature reviews

The analysis of the academic and nonacademic literature on health research impact and multistakeholder initiatives informed the development of an initial database of health research impact indicators.

The extensive body of literature on impact frameworks suggests various dimensions, measures and indicators for evaluating health research. Hence, we conducted a preliminary review of the reviews (i.e. a meta-review), which focused on analysing previous health research impact frameworks [30, 31, 36], and we determined the seven most frequently cited frameworks (i.e. payback model, expected monetary value, Research Impact Framework [RIF], Research Excellence Framework [REF], logic models, Canadian Academy of Health Sciences model, Research Impact Model) that propose indicators and measurement dimensions of health research assessment. The suitability of these frameworks for assessing the impact of multistakeholder initiatives was somewhat limited (e.g. lack of patient engagement and stakeholder participation in defining the indicators, limited coverage of multidimensional impacts or implementation in practice).

Therefore, in addition to the meta-review, we also conducted a literature review on academic and nonacademic research impact studies (not limited to health research) that focused on multistakeholder initiatives between 1992 and 2017. The search yielded 76 articles and reports. For further analysis, we included only the documents that suggested either specific indicators or frameworks for measuring the multifaceted research impact. The final sample consisted of 19 documents for exploring the indicators in more detail. We specifically collected proposed indicators in documents [37–56] that used the established or hybrid forms of the frameworks identified in our meta-review.

The selection of documents was combined with an additional systematic literature review in the Scopus database to gather other specific impact indicators used in practice in health sector organizations and the pharmaceutical industry. This review aimed to identify the commonly used indicators of health sector performance measurement, paying special attention to organizations and the indicators related to measuring the impact of research and innovation activities. We used the following search phrases: (1) “health services” OR “health systems” OR “health research”; (2) “payback”; (3) “indicators” OR “measurement” OR “metrics”. For the pharma industry, the Scopus search continued: (1) “pharmaceutical industry” OR “pharmaceutical sector” OR “pharmaceutical business” OR “pharmaceutical compan*” OR “pharmaceutical firm” OR “pharmaceutical corporation”; (2) “performance assessment” OR “performance measurement” OR “impact”; (3) “indicators” OR “metrics”. For the deeper analysis, we only included studies in English that provided a multidimensional perspective on impact indicators, that is, a broad understanding of the return on investment. We also considered grey literature through a Google search for reports of nonacademic initiatives that contained a specific list of indicators. The final sample consisted of 32 academic articles and 15 nonacademic initiatives.

To identify the potential PRO indicators for the patient-reported dimension (PRD), we conducted a literature review of the current evidence of their usage for research and innovation assessment and a benchmark with relevant existing PRO initiatives such as patient-reported outcome measures (PROMS), PROMOPRO-MS [Profile to Monitor the Progression of Disability in Multiple Sclerosis], and iConquerMS™ [57]. In addition, building on the MULTI-ACT patient engagement guidelines and the related public consultation, we identified a selection of indicators for assessing the performance and effectiveness of patient engagement directly from the patient’s perspective and included this under the PRD. The rationale and methodology of the PRD are detailed in the project’s guidelines and reports (see [58]).

### Indicator analysis for building the database

The 1556 indicators used or suggested for measuring the impact of health research that were identified during the first phase were analysed and combined into a database. Some of the proposed indicators were common or similar among the analyses sources, hence indicating their high relevance for the assessment of health research. All indicators were evaluated on the basis of their meaning and purpose within the framework or initiative in which they were embedded, as well as the intrinsic characteristics of the context for which they were proposed. This analysis allowed us to assess whether and how the indicators’ underlying rationale was in line with the CRIF philosophy.

The payback model taxonomy [37] was chosen as a backbone for the indicator classification, as it is a widely recognized framework for measuring the impact of health research [59, 60] and its categories were easily associated with the CRIF dimensions. The indicators were then classified according to the five CRIF dimensions: excellence, efficacy, economic, social and PROs. They were also sorted according to the stage of the research process to which they were related (input, process, output, outcome, impact). Following an inductive bottom-up approach, we fine-tuned the initial categorization and further grouped the indicators into 53 measurement aspects focused on specific topics within each CRIF dimension (i.e. the overall matter that was assessed by several indicators within the dimension).

In line with the methodology applied to the other four dimensions, the PRD was developed by identifying the most relevant aspects for the specific stakeholder category of “patients” and by selecting the indicators that could measure these identified aspects. All the indicators included under the PRD met the inclusion criteria to be reported by patients (and/or remotely collected) without clinician intervention. The aspects and indicators for the PRD were integrated into the database with the other four dimensions.

### Expert consultation and engagement: semi-structured interviews and focus groups

To collect the perceptions of the stakeholders in the health research domain, we carried out interviews and focus group sessions to engage the stakeholders in the development process of the framework. Based on Concannon et al.’s [61] stakeholder taxonomy, we identified the relevant stakeholder groups: patient and patient organizations, research organizations and funders, policy-makers, the pharmaceutical industry and healthcare providers. The stakeholders were then chosen according to their salience for constructing the CRIF. We analysed a preliminary mapping of 50 potential stakeholders to select those to invite to the strategic working meeting. They had to represent (1) a plurality of perspectives from social to economic and (2) different interests and needs in the health research field, and (3) a strategic importance for the project to achieve its mission, such as policy-makers or patient organizations. The final selection of the most relevant stakeholders (*n* = 12) was made on the basis of deliberative and democratic principles [62] and stakeholders’ attributes [63]. A strategic working meeting helped us identify the different interests of the stakeholders, and the perspectives and dimensions considered important for assessing health research. Moreover, the development of the CRIF had to validate the MSC structure of impact measurement dimensions and aspects in which the indicators were classified.

We also interviewed eight members of the project’s external advisory board (EAB), who represented the following stakeholder groups: academia, patients and patients’ organizations, the pharmaceutical industry, healthcare organizations, health authorities, health innovation and neurodegenerative diseases. Using inductive, semi-structured interviews, we examined the stakeholders’ perceptions of the potential impact indicators and tested the CRIF dimensions. The interviews were held between December 2018 and February 2019, each lasting approximately 1 hour. The interview protocol was flexible, enabling the interviewer to adapt the questions according to the interviewee and the context. It contained 11 core questions, with additional questions to guide the conversation when necessary. The questions covered themes related to the suitability of the CRIF dimensions, as well as the impact aspects and indicators identified in the literature review for the MSC.

The information and feedback received from the interviews and focus groups enabled the refinement of the selection and classification of the indicators identified from the literature reviews. The perceptions of the interviewees and focus groups stressed the importance of including stronger patient and society perspectives when assessing the impacts of research, for example, related to the CRIF dimensions that should consider the benefits for patients and the community, disease development, future research and patients’ daily living, and society at large. From the financial perspective, it was anticipated that improving patients’ quality of life would have broader economic benefits. Concerning the applicability of the framework, we discussed how to ensure the research was in line with the mission, that is, by focusing on the integrating elements of the dimensions; collaboration; and engagement with research partners, professionals and patients in impact assessment.

The feedback of the stakeholders also provided insights for validating the aspects and indicators that are more relevant for evaluating each CRIF dimension. In this regard, to operationalize the MSC, we narrowed down the number of indicators per aspect. For each impact measurement aspect, we selected at least one core and two additional indicators. The core indicators covered matters that were key for evaluating each aspect. They were selected on the basis of the relevance of the topic that they were evaluating for assessing the CRIF dimensions and of the feedback received from the interviewed EAB members. We also classified as core indicators those that were most frequently identified in the literature review for each aspect (for instance, 10 sources suggested that the number of patents was a proxy for evaluating the research outputs within the “intellectual property” aspect of the excellence dimension), assuming that they accounted for relevant matters and the information required for their construction was available to most organizations. Additional indicators complemented the information provided by the core indicators to enable a more comprehensive assessment of each aspect. These indicators were selected because they were often referenced by the analysed sources, regardless of not being the most frequent ones or not covering the most relevant matters for each aspect according to the rationale and values espoused by the CRIF philosophy.

The result of this process was the MSC, comprising a final selection of the 125 most relevant core and additional indicators to measure the multidimensional impact of health research. The MSC was subject to a final validity assessment by the project’s EAB members, by the real case study (Multiple Sclerosis Care Unit) and by an external expert appointed by the European Commission. They provided feedback on suitability and suggested minor improvements (such as gender balance), which we implemented. The next section presents the finalized MSC and its functionalities.

## Results

The MSC transforms the CRIF philosophy into action by providing a wide range of the most relevant indicators evaluating different impact measurement aspects and dimensions. The MSC offers a catalogue of 125 indicators to evaluate the impact of health research. These indicators cover 53 significant measurement aspects related to the five CRIF dimensions: excellence, efficacy, economic, social and PROs. The core indicators (*n* = 54) are expected to be implemented by all the initiatives that intend to apply the framework. Research initiatives can also use additional indicators (*n* = 71) when they consider relevant to enrich the assessment of different aspects and dimensions. Table 1 describes the distribution of indicators per dimension and measurement aspect (see Additional file 1 for details).

**Table 1:** MULTI-ACT MSC and distribution of indicators

Dimensions	Impact aspects	Indicators	Core indicators	Additional indicators
Excellence	20	57	20	37
Efficacy	9	22	9	13
Economic	9	20	9	11
Social	6	15	7	8
Patient-reported	9	11	9	2
Total	53	125	54	71

The MSC contains a description of each indicator, the method of measurement, examples and the type of information needed for each indicator to help research initiatives in their indicator production (see Table 2). In addition to specifying the impact measurement dimension and aspect that an indicator covers, the MSC provides information on how to collect and report data. To inform about different characteristics of each aspect, the MSC includes quantitative (70) and qualitative (40) indicators, as well as others that combine both types of data (17). Out of the 125 indicators, 80 indicators are already used by different organizations, and the MSC provide specific examples of how they are produced.

**Table 2:** Functionalities included in each MULTI-ACT MSC indicator

Information	Description
Dimension	The CRIF dimension to which the indicator relates (excellence, efficacy, economic, social and PROs)
Aspect to be measured	Key topic within each dimension evaluated by the indicator
Type of indicator	Type of indicator within each aspect: core or additional
Description	Content of the indicator
Example	Example from health organizations’ reports or websites
Associated terms	Definition of associated terms that are relevant for understanding the indicator
Comments	Notes that clarify issues related to the computation or use of the indicator
Data type	Overall classification of the type of data the indicator provides: quantitative or qualitative
Expected frequency of data collection	How often data collection is expected
Expected frequency of data dissemination	How often data are expected to be disseminated
Indicator in use	Whether the indicator is currently being used: Yes/No
Limitations	Issues that should be considered when using the indicator
Links	Additional resources that could be helpful in computing and using the indicator
Method of measurement	Description of how the indicator can be measured and reported
Monitoring and evaluation framework	Research stage that the indicator evaluates: input, process, output, outcome, impact
Preferred data sources	Data sources for gathering the data required for elaborating the indicator
Rationale	Relevance of the indicator and advantages of using it
Type of information to be reported	Classification of the information provided in the indicator (e.g. number in physical units, percentage, narrative description)
Unit of measure	If the indicator is quantitative, the units in which the indicator is measured (e.g. number of articles)

Additionally, the MSC is integrated into the MULTI-ACT Digital Toolbox (available at https://toolbox.multiact.eu) together with other CRIF elements. This online platform and the manual (https://toolbox.multiact.eu/node/775) provide more information and examples of the use of the framework and the MSC indicators (see Additional file 2 for details).^1^

One specific feature of the MSC is that the PRD enables the science of patient input, that is, when data on people with a disease are used (active and passive contribution) to evaluate the impact of research and innovation. The PRD includes indicators that are reported by patients, family members and caregivers, addressing functional or psychosocial reported aspects such as PROs and indicators to evaluate the return on engagement. The PROs are investigated as indicators that can measure the outcomes that matter most to patients, and they keep patients and stakeholders engaged along the research and innovation continuum. The PROs are outcomes evaluated directly by the patient and are based on the patient’s perception of a disease and its treatment [64]. These indicators can be a collection of responses to questionnaires, and active or passive data collection without the intervention of clinicians (e.g. e-health via applications or technological devices such as wearables or electronic bracelets).

In the MSC, the PRD reports the perspective of the patient and/or provides continued objective data (e-health), and is therefore not influenced by the clinician. The rationale is that the development of PROs as key indicators of impact is instrumental for enabling a multistakeholder approach and effective patient engagement. PROs are scientifically validated measures reported by patients, which can capture their needs as final beneficiaries of research, thus also capturing the interest of all the health stakeholders.

The MULTI-ACT project delineated a multistakeholder approach, including a set of guidelines [26]. In this context, for a balanced but workable multistakeholder approach it is recommended that multistakeholder research initiatives select a minimum of two indicators per CRIF dimension and a maximum of 12–15 indicators in total. The possibility of aggregating the MSC indicators into a single score to provide an overall assessment of research impact to operationalize the MSC was discussed, but discarded for two reasons. First, the indicators identified across impact measurement aspects and dimensions covered many research performance and impact issues that are incommensurate. Therefore, their aggregation would result in the loss of the richness and specificity needed to evaluate the multidimensional perspective of the return on investment of research. Second, the MSC includes both quantitative and qualitative indicators, thereby preventing aggregation into a single measure. Instead of aggregation, we opted to follow a balanced scorecard approach, which is a widely accepted performance management tool consisting of a “map” of key financial and nonfinancial indicators, as well as four evaluation dimensions (see [65]). This approach is widely applied in business management. Similarly, it consists of a limited number of key indicators to make it manageable while still providing a comprehensive picture of the achievements.

**Fig. 3:**
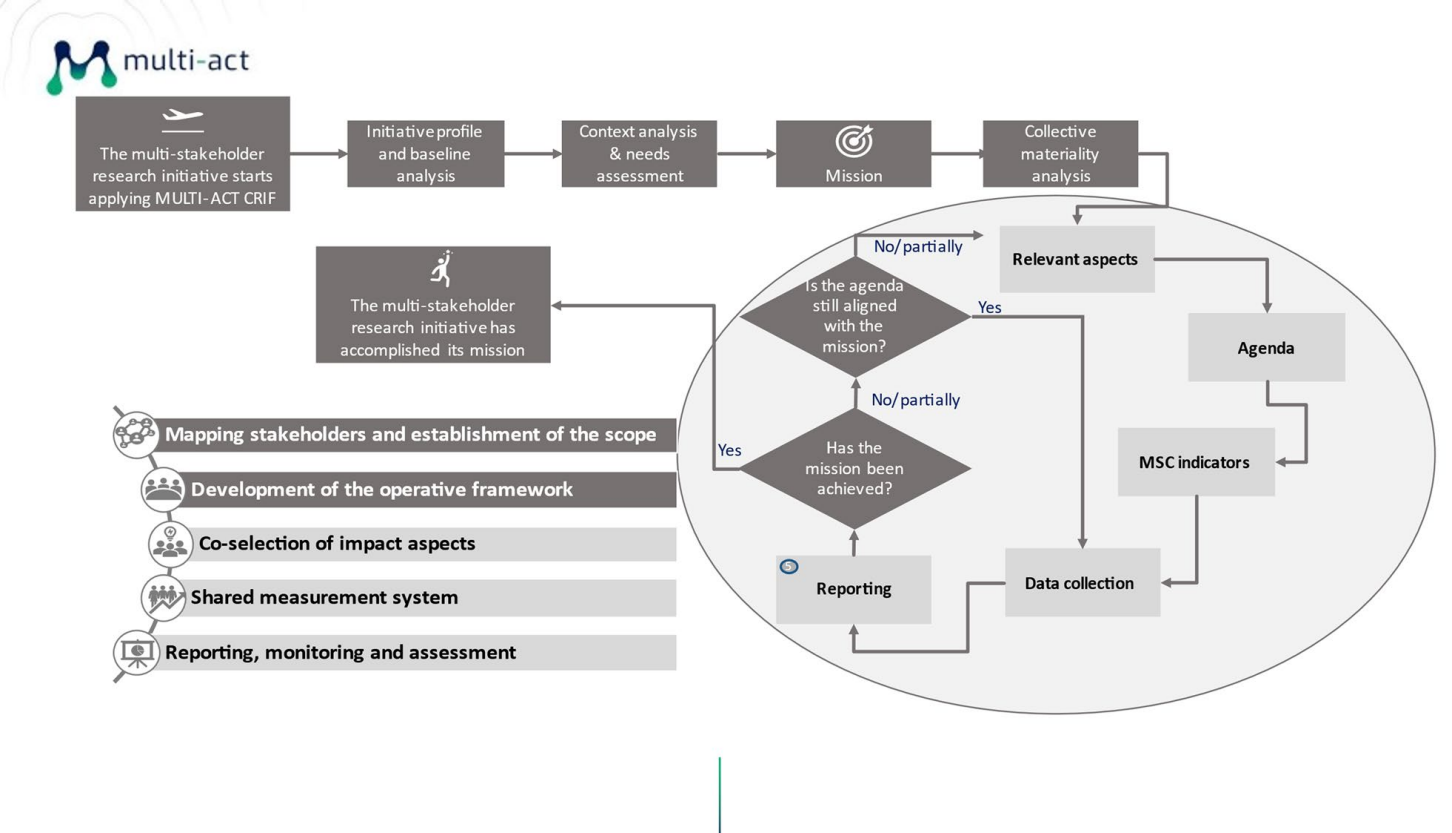
Utilization of MSC as part of CRIF workflow to accomplish the shared mission. The scorecard is relevant for the construction process of a shared measurement system, the co-selection of impact aspects and indicators, and for ensuring that the agenda remains in line with the mission

In practice, multistakeholder research initiatives can identify the most relevant indicators through conducting a collective materiality analysis in the digital toolbox. To accomplish the shared mission, the CRIF workflow includes five main phases, as illustrated in Fig. 3. First, the initiatives must map their key stakeholders and second, together define the mission and operating framework. The stakeholders express their judgments on which impact measurement aspects and indicators in MSC they consider material for the initiative’s mission and agenda. This co-selection process helps articulate an agenda that will act as the basis for the shared measurement system. Finally, the reporting and monitoring of the selected MSC indicators provide the basis for corrective actions to ensure that the agenda is in line with the mission. A specific tool has been developed for each of the phases, and can be found in the digital toolbox.

## Discussion

The assessment of the impact of multistakeholder health research and innovation initiatives in line with the RRI principles is still in its infancy. Most studies have relied on researchers’ views or peer review to assess impacts, instead of involving other important research stakeholders or end-users, such as patients (see e.g. [1, 2, 11, 13]). This seems to have been a methodological weakness of previous frameworks, leaving the research impact assessment process incomplete. Against this background, in this paper, we provided insights into the developmental process of one impact evaluation tool and how MSC, as part of the CRIF, can be used for assessing future RRI measures collectively to push the RRI agenda forward. The MSC is a workable tool that helps gather and respond to diverse stakeholders’ demands. It demonstrates that it is possible to integrate multiple perspectives and measurement aspects. The MSC offers a selection of potential indicators classified under different impact measurement dimensions such as excellence, efficacy, social, economic and PROs. In doing so, the chosen set of indicators is expected to facilitate reaching the shared mission, stakeholder engagement and their return on investment, in whatever form this might take.

Previous health research impact frameworks proposing impact assessment dimensions and various indicators of health research (e.g. [30, 31, 36]) have been limited in covering multidimensionality in impact evaluation, responsiveness to multiple stakeholders’ claims or a narrow understanding of the returns on research, mainly emphasizing academic or financial outcomes (see e.g. [1, 2, 11, 13]). Comprehensive understanding and measurement of the impact of research has remained largely unexplored. One innovative feature of the CRIF, in contrast to existing frameworks, is its mission orientation being tailored for multiple accountability purposes. The mission-related dimension, that is, the efficacy of research, is considered a key element for achieving the shared mission and collective impact [3, 35]. Impact assessment is based on collective decision-making, respecting multiple interests, and placing stakeholders and patient engagement at the core of the impact assessment process. Therefore, the framework could be applied to all types of research initiatives that need broader multistakeholder involvement in and contribution to health research assessment [26]. Each research initiative must define its mission and an operating framework for its realization. The MSC offers varied impact measurement dimensions and aspects that facilitate the co-selection of relevant impact indicators for ensuring that the agenda is in line with the shared mission. Active engagement of all stakeholders throughout the research process encourages initiatives and institutions to apply participatory governance for maximizing social impact.

The European Union (EU) has systematically promoted efforts to integrate societal values into research policy and improve the implementation of RRI [19]. Although studies of RRI implementation have been published [66], the operationalization and institutionalization of RRI principles are still in progress (see e.g. [17, 21, 25]) and initiatives have lacked practical tools for demonstrating the involvement and contribution of patients and society. The engagement efforts of stakeholders and the multifaceted indicators of the MSC, especially the PRD, enable the informational needs and feedback of patients to be captured. Moreover, the MSC helps identify accounting mechanisms for evaluating the health research outcomes that matter most to patients and society. In terms of EU-level implications, the CRIF and MSC have been developed and funded under the Horizon 2020 RRI programme to strengthen RRI institutional change. Moreover, the development process of MSC was multistakeholder in nature, consistent with RRI, and combined research methodologies and stakeholder engagement. The MSC complements other existing responsive innovation initiatives such as the United States Patient-Centered Outcomes Research Institute (PCORI), highlighting the need for validated measures to assess engagement processes and returns on research from multiple perspectives.

For future multistakeholder initiatives, our study shows how the MSC can be applied and developed as part of a collective research assessment effort. It enables multistakeholder initiatives to be co-accountable for the social impact by taking all stakeholders’ informational needs into account when measuring the phenomena (see [33]). As all stakeholders are engaged in identifying and selecting the most relevant impact aspects for the mission of the initiative, the impact assessment is based on considering the plurality of interests.

The main contribution of the study is that it facilitates the evaluation of RRI principles and engages multiple stakeholders in measuring the impacts of health research. The results indicate that making stakeholders co-accountable for research actions is an essential element for achieving broader social impact. To promote the inclusiveness and social responsibility of research, multiple stakeholders should be engaged in decision-making and be able to select the indicators that are the most relevant for them.

One policy implication is that the MSC can be used as an instrument of participatory governance, as it supports collective decision-making processes, and encourages communication on impact aspects and continuous learning. Joint dialogue on relevant indicators will improve the understanding of stakeholders’ multiple interests and their shared mission, thus helping set goals and define accountabilities. At the same time, this process serves to advance the engagement and commitment of stakeholders to working together. Second, the MSC can be exploited in managing health research and innovation initiatives, identifying their research outcomes or controlling their performance. Instead of providing a “score” or “ranking”, the MSC provides information for and feedback from all stakeholders. The final output will help initiatives evaluate their RRI actions and thus understand the multiple impacts that research may produce. The managerial implication of the study is that potential MSC users such as research organizations, institutions and research funders can apply it during different phases and to serve different purposes (see Table 3): at the beginning of the project for planning, during the project for monitoring, and at the end for assessing outcomes. The MULTI-ACT digital toolbox is freely accessible and functional online (available at https://toolbox.multiact.eu).

**Table 3:** Applying MULTI-ACT MSC at different phases

Initiation	Planning: The CRIF dimensions and the potential indicators enable the research initiative to strategically design and evaluate (ex ante) the expected impact of research, in line with its vision and agenda.
Execution	Monitoring: The MSC can serve to implement the shared mission of the initiative. It can be used as a monitoring tool to assess the RRI activities. It can also be used iteratively during the execution of the initiative.
Evaluation and feedback	Assessment: The MSC can be applied at the end of the initiative to assess *how* the desired results were reached. If the MSC is applied from the beginning of the project, the impacts can be compared with the initial evaluation output. This can also help strategically orient future initiatives.

The development of the MSC has some limitations. First, the selection of its indicators may have been related to conceptualization, that is, data collection methods and operationalization of the impact, which is often a very limiting and complex process. Second, the selection of experts for the stakeholder consultation led to focusing on brain diseases and/or expertise in their national or institutional context. A third possible limitation is the short time frame, as the initial version of the MSC was developed during the first phase of the project (2018–2019). Nevertheless, the fact that the MSC was conceived as dynamic, open and customizable may counteract this limitation. Different institutions and research and funding organizations are already putting the CRIF into practice (see for more details [26]), but it is too early to report the outcomes or experiences. Future studies could enrich our study by illustrating the effectiveness of MSC in use, providing best practices or presenting a comprehensive analysis of framework application. Thus, the potential of this practical tool to create an institutional change in RRI, as was the ultimate goal of EU Horizon 2020 programme [25], could be examined. We encourage European funding organizations to require multistakeholder initiatives to test and collectively set a minimal number of indicators for their agenda and to demonstrate their mission achievement using multiple dimensions.

## Conclusions

Our study presents the MSC as an example of how the views of society are a critical starting point in developing new indicators to assess research impacts. Overall, this collective evaluation of research impacts enables the perspective to be extended from the traditional performance and short-term financial return on investment to the demands of RRI and the impacts on society at large. Multidimensionality in impact assessment may make research and innovations more responsive to systemic challenges and develop more equitable and sustainable health services. From the policy development viewpoint, the presented framework is useful for multistakeholder initiatives aiming to facilitate collaborative decision-making in the design of policies, agendas, funding programmes and evaluation procedures or to increase the impact of research on people and society.

## Supplementary Information

**Additional file 1.** Distribution of core and additional indicators per dimension and measurement aspect in MULTI-ACT Master Scorecard as in Digital Toolbox.

**Additional file 2.** Examples of indicators extracted from the MULTI-ACT Toolbox.

## Data Availability

The dataset supporting the results of this article is available from the MULTI-ACT repository, at https://www.multiact.eu. The presented framework and scorecard are freely accessible in the MULTI-ACT Digital Toolbox at https://toolbox.multiact.eu/.

## Abbreviation

CRIF: Collective Research Impact Framework
MSC: MULTI-ACT Master Scorecard
PRD: Patient-reported dimension
PRO: Patient-reported outcomes
RRI: Responsible research and innovation

## Footnotes

1 As the Digital Toolbox is a living tool, it has been recently optimized. The aspects of PRD concerning “return on engagement” have been merged into the single one, and the indicator of the Montreal Cognitive Assessment (MoCA) has been added under the aspect “cognitive function”. Thus, the current PRD includes 8 aspects and 12 indicators (10 core and 2 additional), the MSC 52 and 126, respectively.

## References

[1] Cohen G, Schroeder J, Newson R, King L, Rychetnik L, Milat AJ (2015). Does health intervention research have real world policy and practice impacts: testing a new impact assessment tool. Health Res Policy Syst.

[2] Sarkies MN, Robinson S, Briffa T, Duffy SJ, Nelson M, Beltrame J (2021). Applying a framework to assess the impact of cardiovascular outcomes improvement research. Health Res Policy Syst.

[3] Zaratin P, Battaglia MA, Abbracchio MP (2014). Nonprofit foundations spur translational research. Trends Pharmacol Sci.

[4] Sarkies MN, Bowles KA, Skinner EH, Haas R, Lane H, Haines TP (2017). The effectiveness of research implementation strategies for promoting evidence-informed policy and management decisions in healthcare: a systematic review. Implement Sci.

[5] Sarkies MN, White J, Morris ME, Taylor NF, Williams C, O'Brien L (2018). Implementation of evidence-based weekend service recommendations for allied health managers: a cluster randomised controlled trial protocol. Implement Sci.

[6] Glasziou P, Straus S, Brownlee S, Trevena L, Dans L, Guyatt G (2017). Evidence for underuse of effective medical services around the world. Lancet.

[7] Rosenberg G. Research excellence framework 2014: manager’s report. 2015:121. https://www.ref.ac.uk/2014/media/ref/content/pub/REF_managers_report.pdf. Accessed 8 Oct 2021.

[8] Donovan C, Butler L, Butt AJ, Jones TH, Hanney SR (2014). Evaluation of the impact of National Breast Cancer Foundation-funded research. Med J Aust.

[9] Assasi N, Tarride JE, O'Reilly D, Schwartz L (2016). Steps toward improving ethical evaluation in health technology assessment: a proposed framework. BMC Med Ethics.

[10] Zaratin P, Comi G, Coetzee T, Ramsey K, Smith K, Thompson A (2016). Progressive MS alliance industry forum: maximizing collective impact to enable drug development. Trends Pharmacol Sci.

[11] Ng J, Scahill S, Harrison J (2018). Stakeholder views do matter: a conceptual framework for medication safety measurement. J Pharm Health Serv Res.

[12] Woodland L, Blignault I, O'Callaghan C, Harris-Roxas B (2021). A framework for preferred practices in conducting culturally competent health research in a multicultural society. Health Res Policy Syst.

[13] Pedrini M, Langella V, Battaglia MA, Zaratin P (2018). Assessing the health research’s social impact: a systematic review. Scientometrics.

[14] European Commission. Responsible research and innovation: Europe’s ability to respond to societal challenges, DG research and innovation. Brussels: European Commission; 2012:4. https://op.europa.eu/en/publication-detail/-/publication/2be36f74-b490-409e-bb60-12fd438100fe. Accessed 8 Oct 2021.

[15] Owen R, Pansera M, Simon D, Kuhlmann S, Stamm J, Canzler W (2019). Responsible innovation and responsible research and innovation. Handbook on science and public policy.

[16] Owen R, Macnaghten P, Stilgoe J (2012). Responsible research and innovation: from science in society to science for society, with society. Sci Public Policy.

[17] Silva HP, Lehoux P, Miller FA, Denis JL (2018). Introducing responsible innovation in health: a policy-oriented framework. Health Res Policy Syst.

[18] Weckert J, Valdes HR, Soltanzadeh S (2016). A problem with societal desirability as a component of responsible research and innovation: the “If we don’t somebody else will” argument”. NanoEthics.

[19] Novitzky P, Bernstein MJ, Blok V (2020). Improve alignment of research policy and societal values. Science.

[20] Yaghmaei E (2018). Responsible research and innovation key performance indicators in industry. J Inf Commun Ethics Soc.

[21] De Saille S (2015). Innovating innovation policy: the emergence of ‘Responsible Research and Innovation’. J Responsible Innov.

[22] Morton S (2015). Progressing research impact assessment: a ‘contributions’ approach. Res Eval.

[23] Silva HP, Lefebvre AA, Oliveira RR, Lehoux P (2021). Fostering responsible innovation in health: an evidence-informed assessment tool for innovation stakeholders. Int J Health Policy Manage.

[24] Wickson F, Carew A (2014). Quality criteria and indicators for responsible research and innovation: learning from transdisciplinarity. J Responsible Innov.

[25] Delaney N, Iagher R. Institutional changes towards responsible research and innovation Achievements in Horizon 2020 and recommendations on the way forward. 2020. https://op.europa.eu/en/publication-detail/-/publication/582ef256-cbcc-11ea-adf7-01aa75ed71a1.

[26] Zaratin P, Bertorello D, Guglielmino R, Devigili D, Brichetto G, Tageo V (2022). The MULTI-ACT model: the path forward for participatory and anticipatory governance in health research and care. Health Res Policy Syst.

[27] EU Horizon. Responsible research & innovation. 2020. https://ec.europa.eu/programmes/horizon2020/en/h2020-section/responsible-research-innovation.

[28] European Commission, Directorate-General for Research and Innovation, Indicators for promoting and monitoring responsible research and innovation: report from the Expert Group on policy indicators for responsible research and innovation. Publications Office. 2015. 10.2777/9742. Accessed 21 June 2021.

[29] Adam P, Ovseiko PV, Grant J, Graham KE, Boukhris OF, Dowd AM (2018). ISRIA statement: ten-point guidelines for an effective process of research impact assessment. Health Res Policy Syst.

[30] Milat AJ, Bauman AE, Redman S (2015). A narrative review of research impact assessment models and methods. Health Res Policy Syst.

[31] Raftery J, Hanney S, Greenhalgh T, Glover M, Blatch-Jones A (2016). Models and applications for measuring the impact of health research: update of a systematic review for the Health Technology Assessment programme. Health Technol Assess.

[32] Kok MO, Schuit AJ (2012). Contribution mapping: a method for mapping the contribution of research to enhance its impact. Health Res Policy Syst.

[33] Costa E, Pesci C (2016). Social impact measurement: why do stakeholders matter?. Sustain Account Manage Policy J.

[34] Mazzucato M, Li HLA (2021). Market shaping approach for the biopharmaceutical industry: governing innovation towards the public interest. J Law Med Ethics.

[35] Mazzucato M. Mission-oriented research & innovation in the European Union. A problem-solving approach to fuel innovation-led growth. Brussels: European Commission. 2018. https://ec.europa.eu/info/sites/info/files/mazzucato_report_2018.pdf. Accessed 8 Oct 2021.

[36] Rivera SC, Kyte DG, Aiyegbusi OL, Keeley TJ, Calvert MJ (2017). Assessing the impact of healthcare research: a systematic review of methodological frameworks. PLoS Med.

[37] Buxton M, Hanney S (1996). How can payback from health services research be assessed?. J Health Serv Res Policy.

[38] Buxton M, Hanney S, Morris S, Sundmacher L, Mestre-Ferrandiz J, Garau M et al. Medical research: what's it worth? Estimating the economic benefits from medical research in the UK. Rand.org. 2008. https://www.rand.org/pubs/external_publications/EP20080010.html. Accessed 8 Oct 2021.

[39] Kuruvilla S, Mays N, Pleasant A, Walt G (2006). Describing the impact of health research: a Research Impact Framework. BMC Health Serv Res.

[40] Ovseiko PV, Oancea A, Buchan AM (2012). Assessing research impact in academic clinical medicine: a study using Research Excellence Framework pilot impact indicators. BMC Health Serv Res.

[41] Weiss AP (2007). Measuring the impact of medical research: moving from outputs to outcomes. Am J Psychiatry.

[42] Liebow E, Phelps J, Van Houten B, Rose S, Orians C, Cohen J (2009). Toward the assessment of scientific and public health impacts of the National Institute of Environmental Health Sciences Extramural Asthma Research Program using available data. Environ Health Perspect.

[43] CAHS Making an impact: a preferred framework and indicators to measure returns on investment in health research-Canadian Academy of Health Sciences | Académie canadienne des sciences de la santé. 2009. https://cahs-acss.ca/wp-content/uploads/2011/09/ROI_FullReport.pdf. Accessed 8 Oct 2021.

[44] Banzi R, Moja L, Pistotti V, Facchini A, Liberati A (2011). Conceptual frameworks and empirical approaches used to assess the impact of health research: an overview of reviews. Health Res Policy Syst.

[45] Brueton VC, Vale CL, Choodari-Oskooei B, Jinks R, Tierney JF (2014). Measuring the impact of methodological research: a framework and methods to identify evidence of impact. Trials.

[46] Deloitte Access Economics. Extrapolated returns on investment in NHMRC medical research. Canberra: Australian Society for Medical Research. 2012. https://asmr.org.au/wp-content/uploads/library/ExtrapolatedNHMRC12.pdf. Accessed 8 Oct 2021.

[47] Derrick GE, Haynes A, Chapman S, Hall WD (2011). The association between four citation metrics and peer rankings of research influence of Australian researchers in six fields of public health. PLoS ONE.

[48] Franks AL, Simoes EJ, Singh R, Sajor GB (2006). Assessing prevention research impact: a bibliometric analysis. Am J Prev Med.

[49] Kwan P, Johnston J, Fung AY, Chong DS, Collins RA, Lo SV (2007). A systematic evaluation of payback of publicly funded health and health services research in Hong Kong. BMC Health Serv Res.

[50] Milat AJ, Laws R, King L, Newson R, Rychetnik L, Rissel C (2013). Policy and practice impacts of applied research: a case study analysis of the New South Wales Health Promotion Demonstration Research Grants Scheme 2000–2006. Health Res Policy Syst.

[51] National Institutes of Health (1993). Cost savings resulting from NIH research support.

[52] Sarli CC, Dubinsky EK, Holmes KL (2010). Beyond citation analysis: a model for assessment of research impact. J Med Libr Assoc.

[53] Spoth RL, Schainker LL, Hiller-Sturmhöefel S (2011). Translating family-focused prevention science into public health impact. Alcohol Res Health.

[54] Sullivan R, Lewison G, Purushotham AD (2011). An analysis of research activity in major UK cancer centres. Eur J Cancer.

[55] Taylor J, Bradbury-Jones C (2011). International principles of social impact assessment: lessons for research?. J Res Nurs.

[56] Wooding S, Hanney S, Buxton M, Grant J. The returns from arthritis research. Volume 1: approach analysis and recommendations. RAND Europe. 2004. https://www.rand.org/pubs/monographs/MG251.html. Accessed 8 Oct 2021.

[57] Brichetto G, Zaratin P (2020). Measuring outcomes that matter most to people with multiple sclerosis: the role of patient-reported outcomes. Curr Opin Neurol.

[58] Bertorello D, Brichetto G, Zaratin P. Deliverable D1.8: report on the integration of Patient Reported Outcomes and perspectives into the Collective Research Impact Framework (CRIF). https://www.multiact.eu/wp-content/uploads/2021/02/MULTI-ACT_D1.8_FISM_20200802_v0.6_compressed.pdf.

[59] Aymerich M, Carrion C, Gallo P, Garcia M, López-Bermejo A, Quesada M (2012). Measuring the payback of research activities: a feasible ex-post evaluation methodology in epidemiology and public health. Soc Sci Med.

[60] Graham KER, Chorzempa HL, Valentine PA, Magnan J (2012). Evaluating health research impact: Development and implementation of the Alberta Innovates—Health Solutions impact framework. Res Eval.

[61] Concannon TW, Meissner P, Grunbaum JA, McElwee N, Guise J-M, Santa J (2012). A new taxonomy for stakeholder engagement in patient-centered outcomes research. J Gen Intern Med.

[62] Chilvers J (2008). Deliberating competence: theoretical and practitioner perspectives on effective participatory appraisal practice. Sci Technol Hum Values.

[63] Mitchell RK, Agle BR, Wood DJ (1997). Toward a theory of stakeholder identification and salience: defining the principle of who and what really counts. Acad Manag Rev.

[64] European Medicines Agency, EMA. Appendix 2 to the guideline on the evaluation of anticancer medicinal products in man. The use of patient-reported outcome (PRO) measures in oncology studies EMA. 2016:18. https://www.ema.europa.eu/en/documents/other/appendix-2-guideline-evaluation-anticancer-medicinal-products-man_en.pdf.

[65] Kaplan RS, Norton DP (1996). The Balance Scorecard: translating strategy into action.

[66] Thapa RK, Iakovleva T, Foss L (2019). Responsible research and innovation: a systematic review of the literature and its applications to regional studies. Eur Plan Stud.

