# Supplementary material for: Collective health research assessment: developing a tool to measure the impact of multistakeholder research initiatives
Source: Health Res Policy Syst. 2022 May 2;20:49. doi: 10.1186/s12961-022-00856-9 (PMC9063051; doi:10.1186/s12961-022-00856-9)
Supplement: Supplementary file 1 — Additional file 1. Distribution of core and additional indicators per dimension and measurement aspect in MULTI-ACT Master Scorecard as in Digital Toolbox. [file 12961_2022_856_MOESM1_ESM.docx]

Additional file 1: Distribution of core and additional indicators per dimension and measurement aspect in MULTI-ACT Master Scorecard as in the Digital Toolbox

| **Dimension/aspect** | **Core** | **Additional** |
| --- | --- | --- |
| ***Patient Reported Dimension*** | ***10*** | ***2*** |
| Anxiety and depression | 1 |  |
| Bladder function | 1 |  |
| Cognitive function | 1 |  |
| Fatigue | 1 |  |
| Locomotion | 1 |  |
| Patient satisfaction | 1 | 2 |
| Return on engagement | 3 |  |
| Upper-limb dexterity | 1 |  |
| ***Economic*** | ***9*** | ***11*** |
| Anti-competitive behaviour | 1 |  |
| Control | 1 | 1 |
| Economic externalities | 1 | 3 |
| Financial performance | 1 | 2 |
| Improvement of health services | 1 |  |
| Intellectual property | 1 |  |
| Market | 1 | 2 |
| Organizational efficiency | 1 | 2 |
| Resources allocated | 1 | 1 |
| ***Efficacy*** | ***9*** | ***13*** |
| Drug supply to patient | 1 | 1 |
| Governance | 1 |  |
| Health service assessment | 1 | 2 |
| Health services and products accessibility | 1 | 2 |
| Healthcare practitioners human capital | 1 | 2 |
| Improvement of health services | 1 | 2 |
| Influence on patient behaviour | 1 | 1 |
| Patient quality of life | 1 | 2 |
| Stakeholder engagement | 1 | 1 |
| ***Social*** | ***7*** | ***8*** |
| Corporate reputation | 1 |  |
| Ethical marketing | 1 |  |
| Labor | 1 |  |
| Labour |  | 3 |
| Political externalities | 1 | 2 |
| Socio-environmental impacts | 2 | 3 |
| Stakeholder engagement | 1 |  |
| ***Excellence*** | ***20*** | ***37*** |
| Academic production | 1 | 2 |
| Anticipatory design | 1 |  |
| Bibliometric | 1 | 2 |
| Communication | 1 | 2 |
| Compliance | 1 | 2 |
| Ethics and integrity | 1 | 2 |
| Financial resources | 1 | 2 |
| Impact evaluation | 1 | 2 |
| Influence on public behaviour | 1 |  |
| Influence on subsequent research | 1 | 2 |
| Informing healthcare practice decision making | 1 | 2 |
| Intellectual property | 1 | 2 |
| Patient engagement & involvement | 1 | 2 |
| Products generated | 1 | 3 |
| Research partnership | 1 | 2 |
| Research recognition | 1 | 1 |
| Researchers' human capital | 1 | 3 |
| Resources allocated | 1 | 2 |
| Scientific input | 1 | 2 |
| Stakeholder engagement | 1 | 2 |
| **Total** | **55** | **71** |
