# Supplementary material for: Collective health research assessment: developing a tool to measure the impact of multistakeholder research initiatives
Source: Health Res Policy Syst. 2022 May 2;20:49. doi: 10.1186/s12961-022-00856-9 (PMC9063051; doi:10.1186/s12961-022-00856-9)

**Additional file 2: Examples of MSC indicators extracted from the MULTI-ACT Toolbox**

(All screenshots were retrieved 8.3.2022 from <https://toolbox.multiact.eu/indicators>)

Example of MULTI-ACT Toolbox interface to navigate through indicators

*
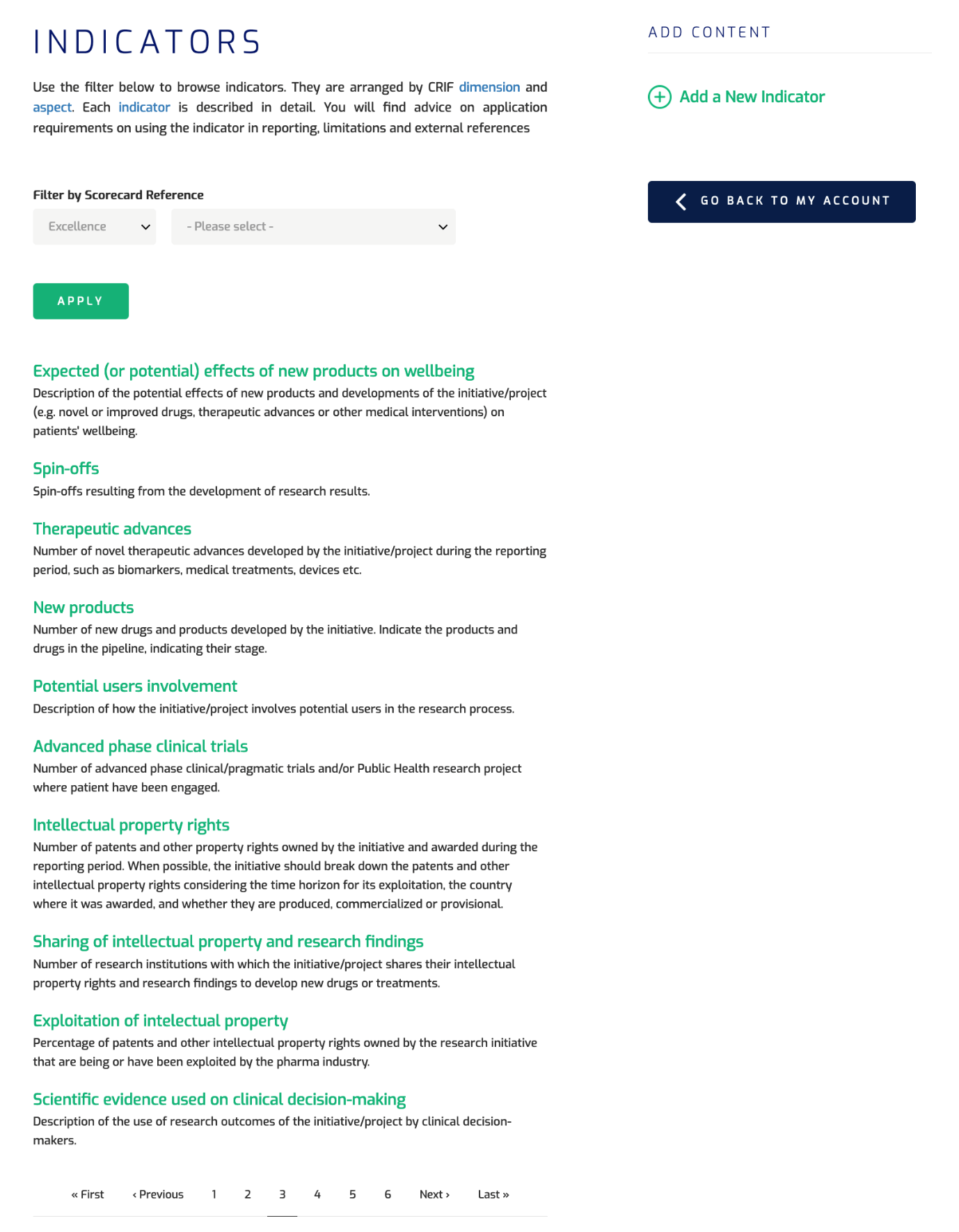
*

Example of information provided on the indicator of “open access publication”


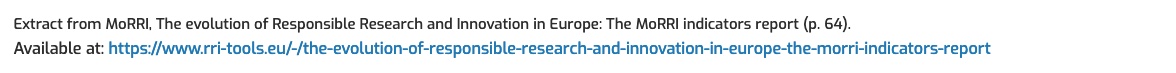

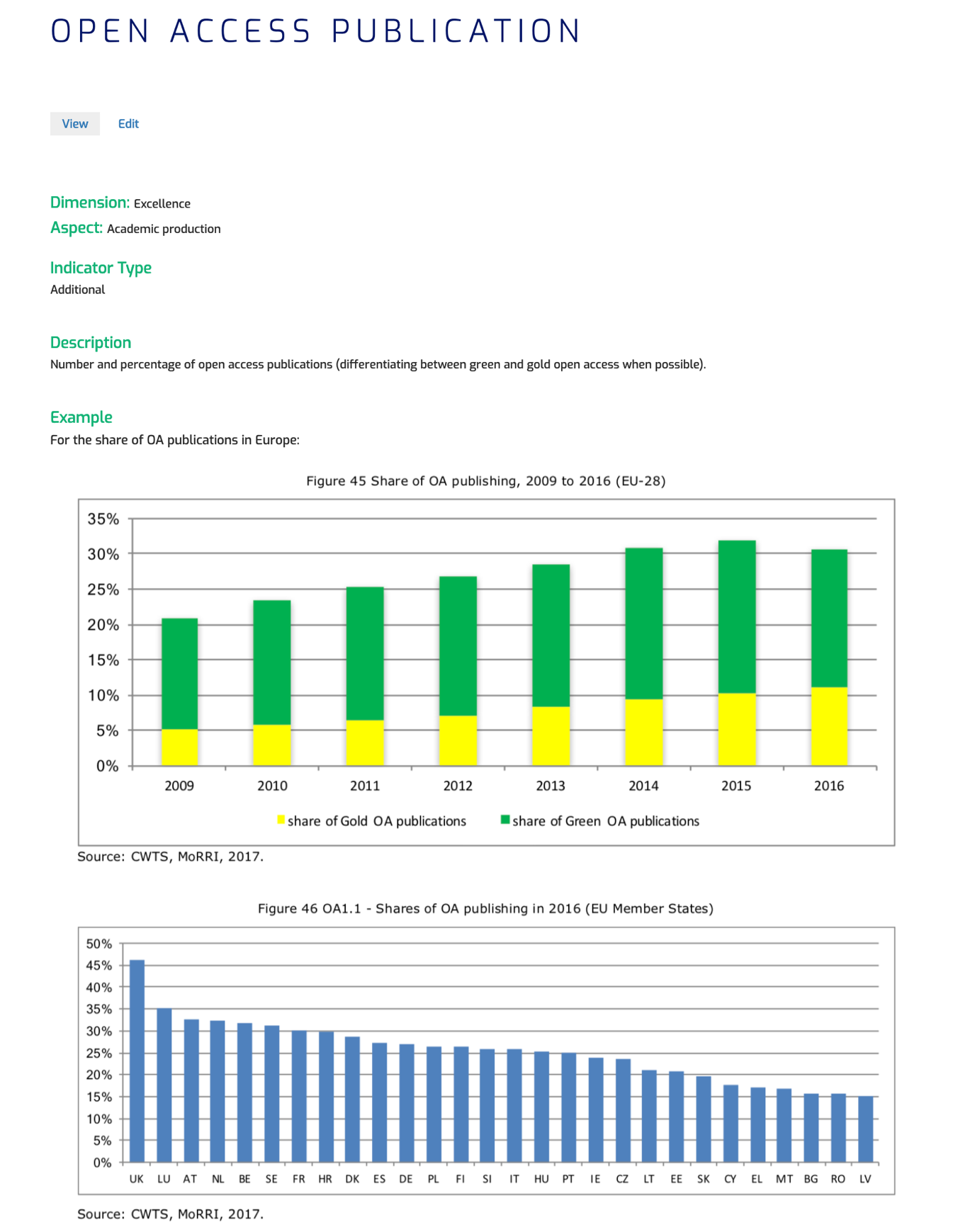


*
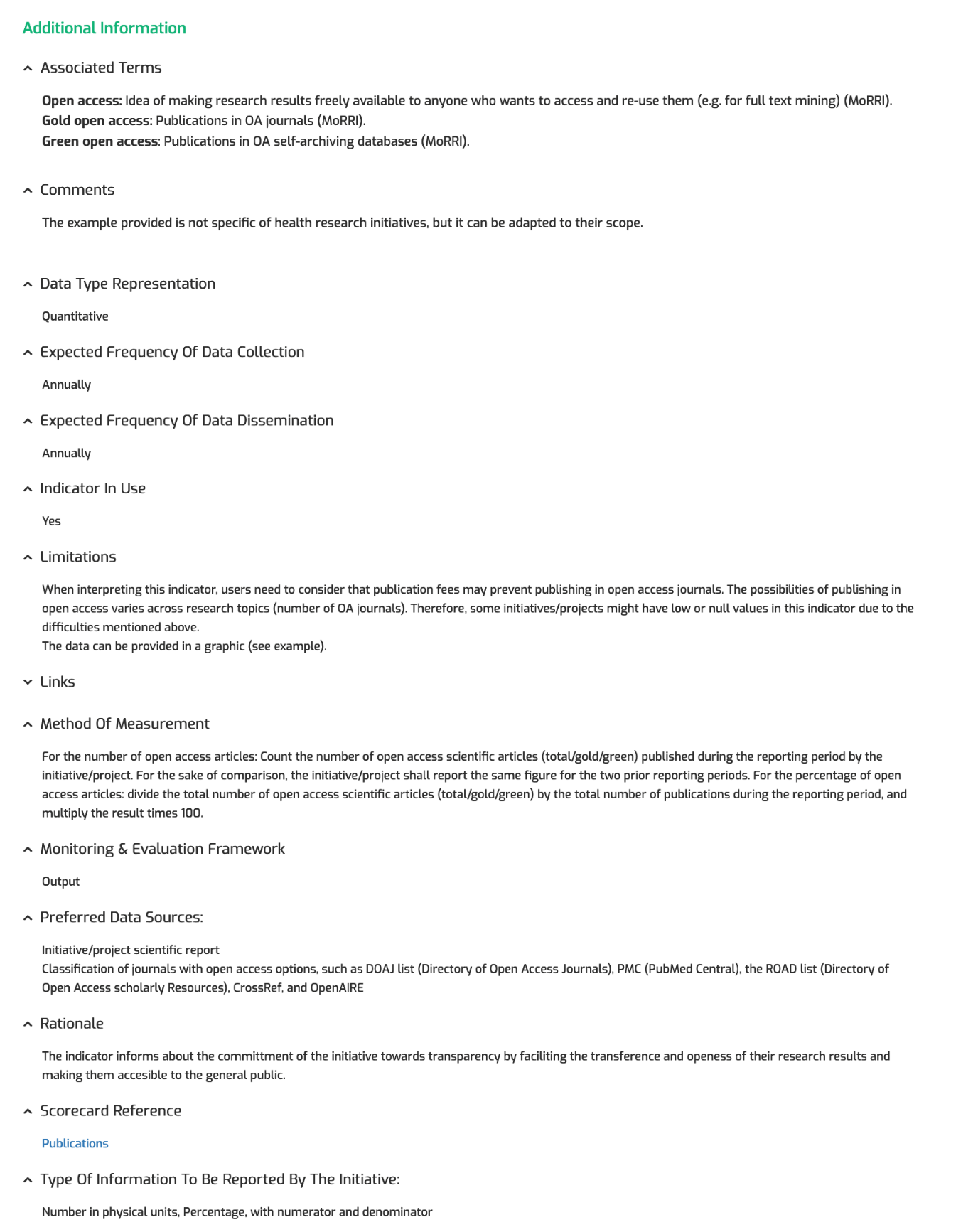
*

Example of information provided on the indicator of “patient engagement”

*
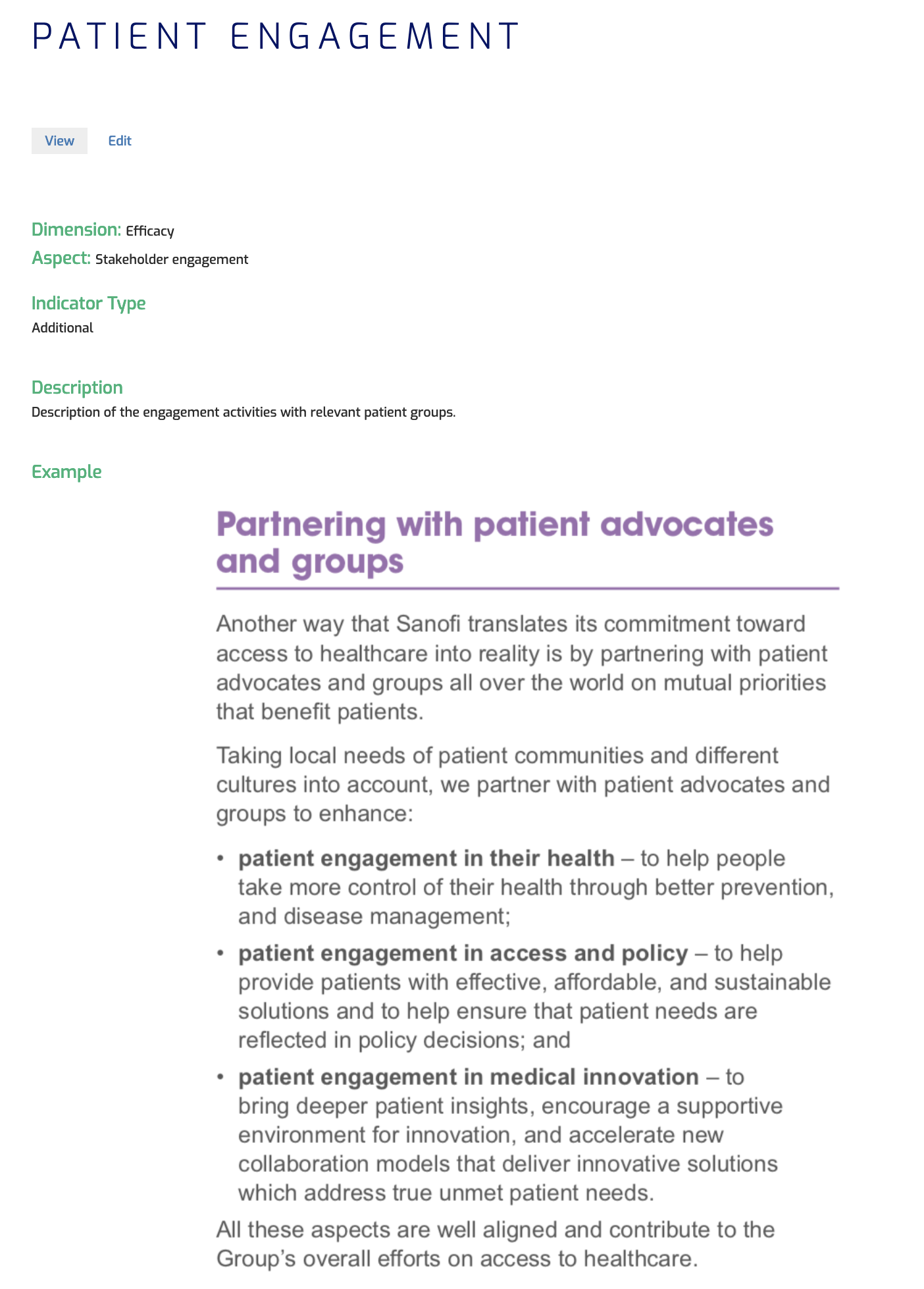
*


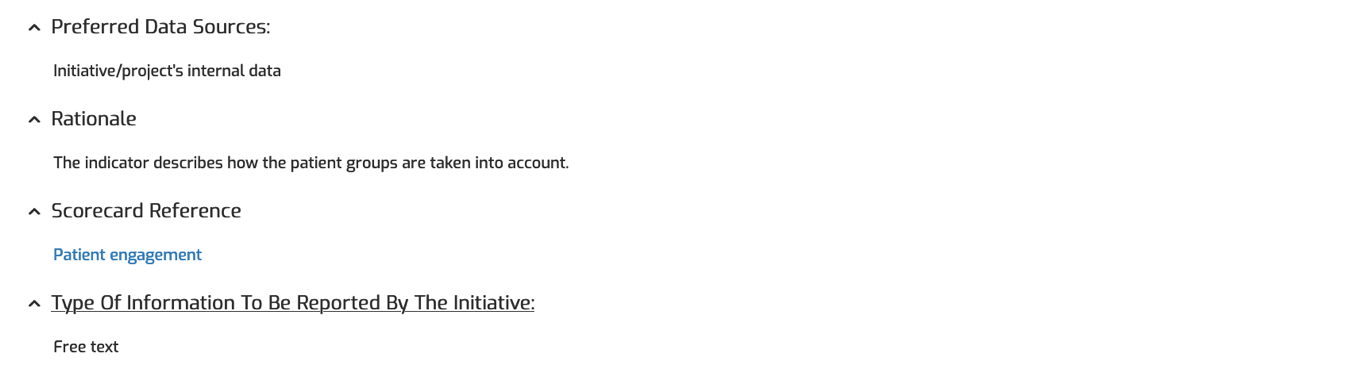

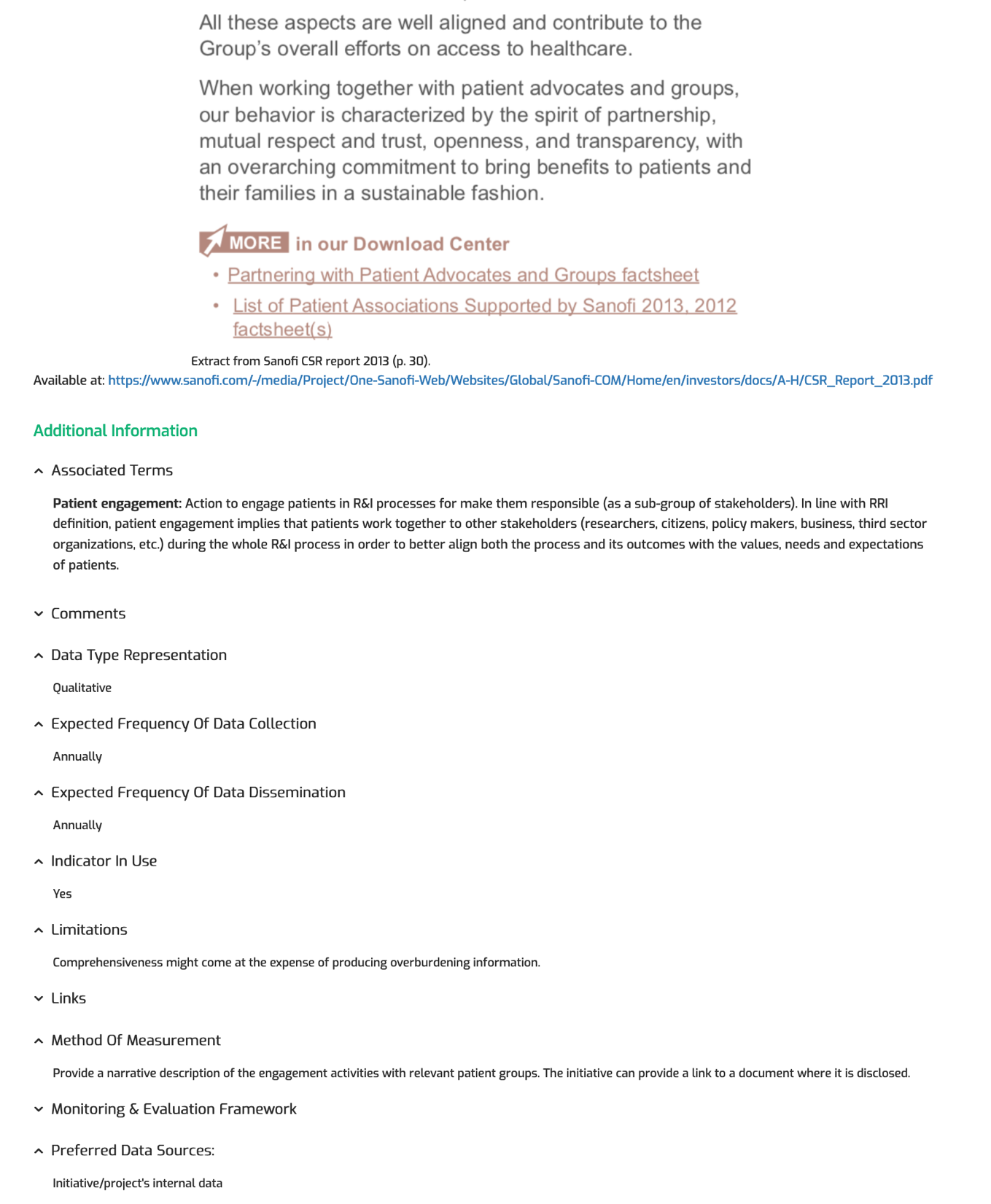

Supplement: Supplementary file 2 — Additional file 2. Examples of indicators extracted from the MULTI-ACT Toolbox. [file 12961_2022_856_MOESM2_ESM.docx]
